# Roles of SMAD and SMAD-Associated Signaling Pathways in Nerve Regeneration Following Peripheral Nerve Injury: A Narrative Literature Review

**DOI:** 10.3390/cimb46070460

**Published:** 2024-07-22

**Authors:** Jeongmin Lee, Dong Keon Yon, Yong Sung Choi, Jinseok Lee, Joon Hyung Yeo, Sung Soo Kim, Jae Min Lee, Seung Geun Yeo

**Affiliations:** 1 Department of Medicine, College of Medicine, Kyung Hee University Medical Center, Seoul 02447, Republic of Korea; sallyljm@khu.ac.kr; 2 Center for Digital Health, Medical Science Research Institute, Kyung Hee University School of Medicine, Kyung Hee University Medical Center, Seoul 02447, Republic of Korea; yondg@khu.ac.kr; 3 Department of Pediatrics, Kyung Hee University School of Medicine, Kyung Hee University Medical Center, Seoul 02447, Republic of Korea; feelhope@khu.ac.kr; 4 Department of Biomedical Engineering, Kyung Hee University, Seoul 02447, Republic of Korea; gonasago@khu.ac.kr; 5 Public Health Center, Danyang-gun 27010, Chungcheongbuk-do, Republic of Korea; joonhyungyeo@gmail.com; 6 Department of Biochemistry and Molecular Biology, College of Medicine, Kyung Hee University, Seoul 02447, Republic of Korea; sgskim@khu.ac.kr; 7 Department of Otorhinolaryngology—Head and Neck Surgery, College of Medicine, Kyung Hee University Medical Center, Kyung Hee University, Seoul 02447, Republic of Korea; sunjaesa@hanmail.net

**Keywords:** SMAD, peripheral nerve injury, degeneration, regeneration

## Abstract

Although several methods are being applied to treat peripheral nerve injury, a perfect treatment that leads to full functional recovery has not yet been developed. SMAD (Suppressor of Mothers Against Decapentaplegic Homolog) plays a crucial role in nerve regeneration by facilitating the survival and growth of nerve cells following peripheral nerve injury. We conducted a systematic literature review on the role of SMAD in this context. Following peripheral nerve injury, there was an increase in the expression of SMAD1, -2, -4, -5, and -8, while SMAD5, -6, and -7 showed no significant changes; SMAD8 expression was decreased. Specifically, SMAD1 and SMAD4 were found to promote nerve regeneration, whereas SMAD2 and SMAD6 inhibited it. SMAD exerts its effects by promoting neuronal survival and growth through BMP/SMAD1, BMP/SMAD4, and BMP/SMAD7 signaling pathways. Furthermore, it activates nerve regeneration programs via the PI3K/GSK3/SMAD1 pathway, facilitating active regeneration of nerve cells and subsequent functional recovery after peripheral nerve damage. By leveraging these mechanisms of SMAD, novel strategies for treating peripheral nerve damage could potentially be developed. We aim to further elucidate the precise mechanisms of nerve regeneration mediated by SMAD and explore the potential for developing targeted nerve treatments based on these findings.

## 1. Introduction

The nervous system of higher animals can be divided into the central nervous system (CNS), consisting of the brain and spinal cord, and the peripheral nervous system (PNS), which innervates the other organs and tissues of the body. The CNS performs higher functions, such as perception and analysis of information, learning and memory, and motor control and emotional regulation, whereas the PNS performs functions such as the receipt and transmission of motor control signals and transmission of sensory information. It is generally the case that nerve cells of the spinal cord or brain fail to regenerate when their axons are injured because of physical or genetic damage, resulting in permanent disability. However, unless the severity of damage to the nerve or cell body is insurmountable, axons of nerve cells in the PNS activate a spontaneous regeneration program and begin regeneration. Thus, there is the potential for these axons to achieve near-complete functional recovery over time [1]. In particular, peripheral nerve fibers can regenerate if the cell body is not damaged. Following the transection of an axon, various anatomical, physical, and chemical changes occur in both proximal and distal segments. In some cases of acute severe damage, there is an initial process of axonal degeneration followed by regeneration. By contrast, axonal degeneration and regeneration may occur simultaneously following chronic compressive injury. Following an axotomy injury, endoneurial fibroblasts and Schwann cells (SCs) proliferate and migrate from the severed nerve fibers, forming a scaffold that bridges the gap. The bands of Büngner, comprising a pillar of SCs, fill the lower endoneurial sheath, creating a passage along which the axon can grow as it descends. Concurrent metabolic changes in the cell body increase the synthesis of RNAs, enzymes, and proteins, fostering axonal regeneration. If axons fail to regenerate, the Büngner bands shrink, SCs decrease, and connective tissue ultimately fills the damaged area [2,3,4].

Many patients suffer from peripheral nerve injury due to crush injuries, trauma, and surgical complications, but no complete treatment exists that can lead to full functional recovery [5]. Clinical treatments for peripheral nerve injury include drug treatments using anti-inflammatory agents such as steroids, surgical treatments, and stem cell therapy. Steroid treatment has various effects such as anti-inflammatory action, immunosuppression, and reduction in edema, but long-term use may cause osteoporosis, diabetes, high blood pressure, and an increased risk of infection [6]. Surgical methods include direct repair, tension-free end-to-end sutures, and autologous nerve grafts. However, these surgical treatments have limitations such as sensory loss, scarring, and neuroma formation at the donor site, and they often fail to achieve complete functional recovery [7,8]. Stem cell therapy using Schwann cells, mesenchymal stem cells, and others offers benefits such as nerve regeneration, neuroprotection, inflammation reduction, immune regulation, angiogenesis promotion, and tissue repair. This field is currently being actively studied, but it also has disadvantages such as neuropathic pain, immune response, tumor formation, risk of infection, and high cost, and it cannot achieve complete functional recovery of peripheral nerves [9,10,11].

This study focuses on SMAD, a key factor closely related to nerve regeneration, and explores the possibility of developing new treatments using it. SMAD plays an important role in nerve regeneration and injury pathways. If a substance that induces complete nerve regeneration by promoting or suppressing the expression of SMAD is developed, it will be possible to overcome the limitations of existing nerve treatments and achieve a groundbreaking breakthrough in the complete functional recovery of patients with peripheral nerve injury.

## 2. Methods

SMADs (Suppressor of Mothers Against Decapentaplegic homologs)—pivotal mediators in transforming growth factor-beta (TGF-β) signaling pathways—orchestrate fundamental cellular processes, including growth, differentiation, and apoptosis. Although research progress has been made on the role of SMADs in various diseases, their roles in nerve degeneration and regeneration after nerve injury remain poorly understood. To provide a summary of current knowledge on the subject, we conducted a literature review of studies examining SMAD involvement in recovery from peripheral nerve injury and effects of electrical stimulation, published between January 1998 and March 2024. Studies were retrieved from five electronic databases—PubMed, SCOPUS, Cochrane libraries, EMBASE, and Google scholar—by one of the authors (J.H.Y) based on the search terms, ‘SMAD’, ‘peripheral nerve’, ‘nerve injury’, ‘nerve degeneration’, and ‘nerve regeneration’. Prospective or retrospective animal or human studies on SMAD in peripheral nerves or SMAD-related research on the degeneration and regeneration of nerves after injury were included. Studies were excluded if they fell into any of the following categories: (1) unpublished data, (2) review articles, (3) gray literature, (4) case reports, (5) duplicate cases, or (6) literature not published in English.

## 3. Results

**Figure 1:**
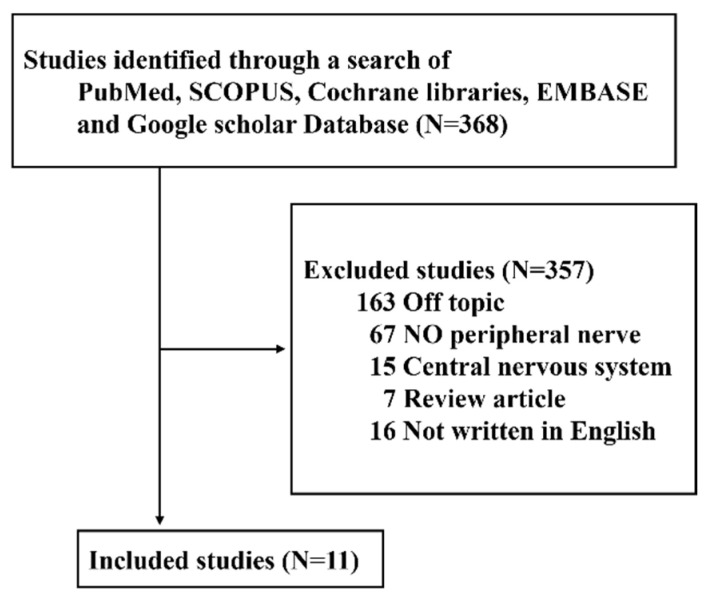
Review flow diagram.

A total of 11 papers related to SMAD expression after peripheral nerve injury satisfied inclusion/exclusion criteria, including seven on the sciatic nerve, three on the dorsal spinal cord, one each on the optic nerve and hypoglossal nerve, and two cell studies (Figure 1). After peripheral nerve injury, SMAD1, -2, -4, and -5 increased, and SMAD8 decreased. In addition, following peripheral nerve injury, SMAD1 and -4 promoted nerve regeneration, whereas SMAD2 and -6 hindered nerve regeneration. SMAD-related signaling pathways related to nerve regeneration after peripheral nerve injury included the BMP/SMAD1, BMP/SMAD4, BMP/SMAD7, and PI3K/GSK3/SMAD1 pathways (Table 1).

## 4. Discussion

### 4.1. Expression of SMADs and Activation of SMAD-Associated Signaling Pathways in Nerve Regeneration and Degeneration after Peripheral Nerve Injury

Following nerve damage, neurons in the PNS can survive and regenerate, but neurons in the CNS cannot. In response to peripheral nerve injury, a diverse array of molecules, including neurotrophins and cytokine receptors such as TrkA, TrkB, cRet, GFRα1, LIFR, and CNTFR α, are upregulated in neurons. The simultaneous induction of these molecules contributes to signal transduction and plays an important role in cell survival and nerve regeneration. Intracellular signaling molecules such as Shc, ERK, PI3K, Akt, JAKs, Tyk, and STAT3, which act downstream of these receptors, are also activated, together with transforming growth factor-β (TGF-β), bone morphogenetic protein (BMP), and activin. Members of the TGF-β superfamily, in particular, have received considerable research attention owing to their proposed role as nerve regeneration factors [23,24].

TGF-β is widely expressed in various tissues and is involved in regulating various physiological processes, playing an important role in embryonic development, tissue regeneration, cell differentiation, and regulation of the immune system. Since the discovery of TGF-β1 in 1982, the TGF-β superfamily has grown to include three TGF-β isotypes (TGF-β1, TGF-β2, TGF-β3), activin, and BMPs. More than 30 structurally similar ligands, including growth differentiation factor (GDF), have been identified to date, making this the single most-studied cytokine family [25,26]. The TGF-β1 signaling process begins with binding of TGF-β to TGF-β receptor II (TGF-βRII), which then forms a dimer with TGF-βRI, leading to phosphorylation and activation of downstream targets, including SMADs [27].

The structure of SMADs consists of an N-domain and a C-domain separated by a middle linker. Both C- and N-terminal domains are characterized by a proline-rich structure, the largest of which is found in SMAD4/DPC4. Individual SMAD proteins are indirectly activated as a result of the interaction of specific ligands with surface receptors. For example, SMAD1 is activated by BMP2 or BMP4 signaling; both SMAD2 and SMAD3 carry out activin and TGF-β signaling; and SMAD4 plays a universal role in mediating these signaling pathways [28,29].

SMADs are classified into three types according to their structure and function. The first type is receptor-regulated SMADs (R-SMADs), which include SMAD1, -2, -3, -5, and -8. SMAD2 and SMAD3 respond to TGF-β, activins and inhibin, whereas SMAD1, -5, and -8 function in the BMP signaling pathway. Atypically, TGF-β can also activate SMAD1, -5, and -8 in endothelial cells [27,30]. R-SMADs are directly phosphorylated and activated by type I serine/threonine kinase receptors. The second is the common partner type SMAD (Co-SMAD), exemplified by SMAD4. R-SMADs oligomerize with Co-SMADs to form a heterodimeric complex that translocates to the nucleus, where it regulates transcriptional responses. In cells deficient for either R-SMAD or S-SMAD, TGF-β signaling to the nucleus is impaired, highlighting the importance of these two types in nuclear signaling. Because mutual cooperation between the two SMAD types is essential for the operation of this nuclear signaling pathway, the transcription process does not proceed without a Co-SMAD even if the R-SMAD enters the nucleus independently. Despite this, it has been reported that SMAD4 is not absolutely required for the TGF-β signaling pathway [31,32]. The third type comprises the inhibitory SMADs (I-SMADs), SMAD6 and -7, which inhibit SMAD-mediated signal transduction by interacting with type I receptors (R-SMADs) and preventing their activation [33]. I-SMADs are proposed to play an inhibitory role in the TGF-β signaling pathway by directly binding to TGF-βRI and interfering with the binding and activation of R-SMADs; I-SMADs can also combine with Co-SMADs to prevent the formation of SMAD hetero-oligomers [34,35].

### 4.2. Expression and Role of SMAD1–8 in Nerve Regeneration after Peripheral Nerve Injury (Table 1, Table 2 and Table 3)

There have been a number of experimental studies on SMAD expression in relation to nerve regeneration after nerve damage. These are summarized below, classified by nerve.

**Table 2:** SMAD expression in the context of peripheral nerve degeneration and regeneration after nerve injury.

Nerve Injury	Increased/Decreased	SMAD Type	Functions
Transection	Increased	SMAD1 SMAD4	Regeneration
		SMAD2	Degeneration
		SMAD5 SMAD8	Not yet determined
Compression	Increased	SMAD1 SMAD4	Regeneration
		SMAD2	Degeneration
		SMAD5 SMAD8	Not yet determined

**Table 3:** SMAD expression results according to nerve damage and SMAD type.

SMAD Type	Types of Target Nerves and Methods of Nerve Damage	Results
SMAD1	Hypoglossal nerve transection Sciatic nerve transection Dorsal column and sciatic nerve compression Optic nerve compression Retinal ganglion cell transection Transfection of Xenopus embryos with SMAD6	Neuroregeneration
SMAD2	Hypoglossal nerve transection Dorsal column and sciatic nerve compression Sciatic nerve compression	Neuroregeneration
SMAD3	No research	-
SMAD4	Hypoglossal nerve transection Transfection of Xenopus embryos with SMAD6 Dorsal column and sciatic nerve compression Optic nerve compression Retinal ganglion cell transfection	Neuroregeneration
SMAD5	Sciatic nerve transection Dorsal column and sciatic nerve compression Optic nerve compression Retinal ganglion cell transection	Not yet determined
SMAD6	Transfection of Xenopus embryos with SMAD6	Neurodegeneration
SMAD7	No research	-
SMAD8	Hypoglossal nerve transection Sciatic nerve transection Dorsal column and sciatic nerve transection Optic nerve compression Retinal ganglion cell transection	Not yet determined

#### 4.2.1. Hypoglossal Nerve

One qualifying study examined the expression of SMAD1–8 after hypoglossal nerve transection in 42 male Wistar rats aged 7 weeks and compared it with that in the control group 7 days after injury. Reverse transcription-polymerase chain reaction (RT-PCR) and in situ hybridization showed that SMAD1, -2, -3 and -4 mRNAs increased, whereas SMAD8 mRNA decreased in hypoglossal nuclei on the injured side (*p* < 0.01). Immunohistochemistry and Western blot analyses confirmed a corresponding increase in SMAD1, -2, and -4 in ipsilateral injured motor neurons in hypoglossal nucleus (*p* < 0.01). The authors proposed that SMAD-mediated signaling plays an important role in the process of nerve regeneration after nerve injury [12].

#### 4.2.2. The Dorsal Root and Dorsal Column of the Spinal Cord

Two qualifying studies investigated SMAD involvement in the response to spinal cord injury. In the first, Fisher 344 rats (*n* = 94) were injured by performing a dorsal root transection and dorsal column injury and then divided into four treatment groups—fGFP (green fluorescent protein) treatment only (control); ATF3 (activating transcription factor 3) treatment only; SMAD1 only; and combined treatment with fGFP ATF3, c-Jun, STAT3 (signal transducer and activator of transcription 3) and SMAD1—administered via an adeno-associated viral vector. Axonal regeneration was analyzed immunohistochemically on weeks 8, 10, and 20 after injury and compared among groups. Regeneration occurred most rapidly in ATF3-only and combination groups compared with controls (*p* < 0.05), and there was no significant difference in recovery rate between the ATF3-only and combination groups. These studies implicated SMAD1 in nerve regeneration, but found that co-treatment with ATF3, c-Jun, and STAT3 was not superior to SMAD1 administration alone, indicating the absence of a synergistic effect [13]. In the second study, the sciatic nerve and ascending sensory fibers of the right sciatic nerve of the spinal cord of C57BL/6 mice (*n* = 10) were transected as part of an in vivo and in vitro study. Acetyl-histone H4 (AcH4) modifications in the promoters of SMAD1, Atf3, Sprr1a, Npy, and Galantin in DRG neurons, measured by Western blot analysis 6 h after peripheral axotomy or sham surgery, were significantly decreased in the injured compared with non-injured DRG neurons (SMAD1, Atf3, *p* < 0.001; others, *p* < 0.05). As nerve regeneration began in DRG neurons, H4 acetylation was restored, and the repertoire of regeneration-associated genes was transcribed, followed by an increase in SMAD1 expression [14].

#### 4.2.3. Sciatic Nerve

One study employing a sciatic nerve-crush injury model compared axon regeneration following injury in wild-type mice (controls) and mice lacking PROM1, a regulator of the axon regeneration program. Following the nerve crush injury, axon regeneration was reduced by ~30% in Prom1-KO mice compared with that in the control group, and the amount of phosphorylated SMAD2 (p-SMAD2) was also reduced. Overexpression of PROM1 in cultured DRG neurons increased the amount of p-SMAD2 and promoted axon regeneration in vitro. From this, the authors inferred that PROM1 plays an important role in activating the neuronal regenerative program and further concluded that this process is closely related to SMAD signaling [15].

Another study examined changes in SMAD1, -5, and -8 expression in both humans and mice. In a cohort study using human ALS (amyotrophic lateral sclerosis) muscle samples (27 patients, 33 controls), qRT-PCR showed that the expression of SMAD8 mRNA increased approximately 19-fold compared to that in the control group (*p* < 0.0001), whereas SMAD5 (~3-fold) and SMAD1 mRNA showed lesser increases (*p* < 0.05). This pattern of increased expression became more accentuated as the disease progressed. In this same study using a G39A superoxide dismutase mouse model, researchers performed a sciatic nerve transection injury and examined SMAD1, -5, and -8 levels by qRT-PCR 150 days after the injury. SMAD1 and -5 increased by ~5-fold (*p* < 0.01), and SMAD8 increased 17-fold in the injury group compared with the control group (*p* < 0.01), a pattern that showed a stage-dependent increase starting from the presymptomatic stage (60–105 days after injury). Therefore, as the pathology progresses in both human samples and mouse models, the expression of SMAD1, -5, -8 mRNA, and protein increases in neurodegenerative diseases, suggesting that these changes can be used as disease progression markers [16].

### 4.3. Expression and Role of SMAD-Associated Signaling Pathways in Nerve Regeneration after Peripheral Nerve Injury

The major molecular pathways involved in neurite growth and nerve survival include the PI3K (phosphatidylinositol-3 kinase)/Akt (protein kinase B) transporter system, Ras-ERK (rat sarcoma-extracellular signal-regulated kinase) pathway, and cyclic adenosine monophosphate (cAMP)/protein kinase A (PKA) and Rho kinase signaling pathways. The PI3K/Akt transport system appears to be involved in neurotrophy; it also prevents cell death and participates in growth and differentiation. The Ras/ERK pathway plays the most important role in promoting neurite growth and also increases axon survival [36]. The cAMP/PKA pathway has been shown to be important for neuronal growth, survival, and differentiation both in vitro and in vivo [37]. In the Rho kinase neurotransmission pathway, Rho GTPase plays a pivotal role in guiding nerve growth by responding to extracellular ligands [38,39]. The proteoglycan-digesting enzyme, chondroitinase ABC, is a key regulator of this pathway [40], which is also modulated by the pharmacological agents fasudil and ibuprofen.

Given the known role of SMAD1 as a transcriptional regulator that plays an important role in cell growth, differentiation, shape change, and survival by recognizing external signals and regulating the expression of various classes of genes [19], we further explored the literature on pathways related to SMAD signaling.

#### 4.3.1. BMP/SMAD1 Pathway

Using Xenopus embryos and mammalian cells, one study investigated cellular consequences of molecular interactions among SMADs that might inform nerve regeneration processes. In Xenopus experiments, co-injection of SMAD2 and SMAD6 (experimental group) resulted in the formation of secondary axes, which extended more anteriorly compared to the control group. Moreover, SMAD6, overexpressed in mammalian cells through transfection, does not directly interact with the C-domain of SMAD2 or SMAD4. Instead, it competitively binds to the C-domain of SMAD1 in the presence of BMP2 stimulation, inhibiting the formation of the SMAD1-SMAD4 complex. The authors of this report concluded that SMAD6, as an antagonist of the BMP/SMAD1 pathway, plays a suppressive role in the nerve regeneration process [17].

In a second study conducted in vivo, C57BL/6 mice genetically modified to knock out SMAD1 were divided into three groups—DC, SN, and preconditioned SN (pSN) + DC—where DC and SN denote dorsal column (DC) transection and sciatic nerve (SN) transection, respectively. Ten days after performing transections, changes in gene expression and axon regeneration were analyzed and compared among groups. After injury, mRNA levels of SMAD1, SMAD2, SMAD4, SMAD5, SMAD5, and Bmp4 were upregulated in both SN (2-fold) and pSN + DC (~7-fold) groups compared with the DC group (*p* < 0.001). An immunohistochemical analysis of SMAD1, BMP4, and Noggin protein levels showed that BMP4 and SMAD1 were upregulated in pSN + DC and SN groups, whereas Noggin was upregulated in DC and sham groups. To test the functional role of BMP4 and SMAD1 upregulation, these researchers stimulated BMP4 signaling (which acts upstream to activate SMAD1) through the overexpression of BMP4 using AAV encoding BMP4, and then compared the axonal recovery of experimental and control groups electrophysiologically 1 week later. Axonal growth was significantly increased (by ~60%) in the experimental group compared with the control group (*p* < 0.001). Collectively, these results suggest that the SMAD1-dependent BMP pathway plays an important role in axonogenesis [18]. BMP signaling is required for the initiation and elongation of nerve regeneration in both the CNS and PNS, and BMP4 increases axon growth potential by activating SMAD1. This minimally invasive and clinically applicable AAV-based in vivo gene delivery method can be used to promote axon regeneration via the BMP/SMAD1 pathway.

#### 4.3.2. BMP/SMAD4 Pathway

In a related study designed to explore BMP/SMAD signaling mechanisms in nerve regeneration, adult female Sprague Dawley rats were randomly assigned to five groups: an uninjured intact control group (IC) group; a sham-treated control group (partial laminectomy but no DC crush injury); a non-regenerating DC group (DC), which received a DC lesion alone; a regenerating SN group (SN), which received a SN lesion alone; and a regenerating pre-conditioning SN lesion (pSN) + DC injury group. Afterwards, the role of the BMP4/SMAD1 pathway in nerves was investigated by comparing mRNA and protein expression levels of SMAD1–8, TGFβ1 and -2, and BMP2, -4, and -7 among groups. mRNA levels of SMAD1, SMAD2, SMAD4, SMAD5, SMAD8, and BMP4 were all upregulated 2.0- to 6.7-fold in experimental groups compared with sham controls, whereas Noggin (an endogenous BMP antagonist) was downregulated 2-fold compared with controls. When BMP4 treatment was applied to DRGN cultures, neurite outgrowth was disinhibited despite inhibitory concentrations of CME [19]. These results suggest that the BMP4/SMAD1 signaling pathway promotes DRGN survival and induces nerve regeneration independently of the mTOR pathway. Intra-DRG injection of AAV-BMP4 significantly increased Bmp4 mRNA levels (7.6 ± 0.3-fold) compared with the DC + AAV-Null group, an increase that was accompanied by a significant increase in BMP4 protein levels in the AAV-BMP4–injected group (642 ± 1.2 ng/mg) compared with the DC + AAV-Null group (9.7 ± 1.2 ng/mg). The authors concluded that activation of the BMP4/SMAD1 pathway contributes to nerve regeneration [19].

In vivo and in vitro studies by our group targeting adult rodent retinal ganglion cells (RGCs), we investigated whether the BMP4/SMAD1 intracellular signaling pathway provides neuroprotection. Rats were divided into three groups: intact, ONC (optic nerve crush injury), and ONC + PN (peripheral nerve) graft. In RGCs 21 days after ONC + PN treatment, BMP4, SMAD1, SMAD4, SMAD5, SMAD8, Smif, and Msg1 mRNA levels increased by 3.2-, 4.5-, 5.5-, 2-, 3-, 3-, and 2-fold compared with the control group (*p* < 0.05). Additionally, inhibition of BMPR1a, BMPR1b, ACVR1, and SMAD1 in RGC culture inhibited neurite outgrowth and mean neurite length. These results implicate the activity of the BMP4/SMAD1 pathway in RGC survival and axon regeneration [22]. BMP signaling causes the phosphorylation of SMAD1/5/8, which then form a complex with SMAD4 and move to the nucleus to regulate target gene expression [41]. Exogenous BMP promoted RGC survival by approximately 30% and played a role in promoting RGC neurite outgrowth, which was associated with the activation of SMAD1. Therefore, the BMP4/SMAD1 pathway promotes survival and axon regeneration, and this mechanism can be used as a therapeutic target for nerve damage.

#### 4.3.3. BMP/SMAD7 Pathway

In another study on BMP/SMAD signaling in nerve regeneration, an axonal degeneration model was created in Sprague Dawley rats by applying a sciatic nerve crush injury using an aneurysm clip. BMP binds to BMP type I and type II serine/threonine kinase receptors on the cytoplasmic membrane, activating intracellular SMAD proteins. BMP7, BMP receptors, p-SMAD1, -5, -8, and Noggin expression levels were compared between control and experimental groups at 1, 2, 4, and 8 weeks after injury. Depending on the receptor binding of BMP7, the levels of downstream substances, SMAD1, SMAD5, and SMAD8 proteins, increased following nerve damage and remained elevated for 4 weeks, whereas Noggin was significantly upregulated from 2 to 8 weeks after injury. By comparison, BMP7 and SMAD1, 5, and -8 protein levels were virtually undetectable in the undamaged control group. Interestingly, the application of parathyroid hormone (1–34) to SCs increased the expression of BMP7 compared with the vehicle (saline) control group in association with a significant increase in SCs proliferation. From this, the authors inferred that the BMP7/SMAD pathway plays an important role in peripheral nerve regeneration [20]. Therefore, BMP-7 can be used as a new strategy for nerve damage, as its administration can induce axonal regeneration.

#### 4.3.4. PI3K/GSK3/SMAD1 Pathway

In addition to BMP-related molecular pathways, it has recently been reported that SMAD1, as part of a PI3K/GSK3/SMAD1 signaling pathway, plays an essential role in nerve regeneration. PI3K (phosphoinositide 3-kinase) regulates intracellular signaling processes involved in various functions, including cell growth, proliferation and differentiation, migration, and survival [42,43]. GSK3 (glycogen synthase kinase 3), an enzyme that regulates glucose metabolism, has also been found to play a variety of important roles in the nervous system, including the development and differentiation of nerve cells and regulation of cell death; importantly, it is also closely associated with various neurological diseases [44]. The role of the PI3K/GSK3/SMAD1 signaling pathway in nerve regeneration following dorsal root ganglion (DRG) dissection and sciatic nerve crush injury was tested in 8–10-week-old female CF-1 mice (30–35 g). An analysis of PI3K/GSK3 signaling, SMAD1 expression and axon regeneration 3 h, 2 days, and 3 days after peripheral axotomy showed that PI3K/GSK3 signaling was activated, resulting in increased SMAD1 expression and promotion of axon regeneration. On the basis of these results, the authors reported that the PI3K/GSK3/SMAD1 pathway plays an important role in nerve regeneration [21]. Peripheral nerve injury activates PI3K downstream, and PI3K generates phosphoinositol (3,4,5)-triphosphate (PtdIns(3,4,5)P3), which activates kinases such as PDK1, AKT, and GSK3, inducing a signaling cascade. Among these, GSK3 is a principal regulator of axon regeneration and activates the transcription-dependent axon regeneration program, leading to an increase in SMAD1. SMAD1 is essential for nerve regeneration to the extent that its depletion can prevent axon regeneration. PI3K regulates the activity of GSK3, and additional research is needed to identify the upstream regulators that modulate GSK3 signaling [21].

This study had several limitations. First, all existing studies were animal experiments or laboratory studies; there were no research results on humans. Therefore, future research on humans is needed. Second, the included studies mainly used the sciatic nerve or dorsal root and dorsal column of the spinal cord. Given that SMAD is involved in nerve regeneration or degeneration in various nerves, future research on other nerves is needed. Third, additional research is needed to elucidate the detailed mechanisms underlying the known characteristics of SMAD1 to 8. Fourth, given the various intracellular signaling pathways related to SMAD, studies are needed to identify which signaling molecules promote SMAD in relation to nerve regeneration and which signaling molecules reduce SMAD in relation to neurodegeneration. Fifth, in the future, we hope to contribute to the treatment of patients suffering from neurological diseases by working on the development of drugs or substances that activate SMAD1 and SMAD4, which are related to nerve regeneration, and drugs or substances that inhibit SMAD2 and SMAD6, which are related to neurodegeneration. Sixth, in addition to SMAD, future studies should examine other regenerative factors related to complete nerve recovery after nerve damage.

## 5. Conclusions

Major SMAD signaling pathways related to nerve regeneration include BMP/SMAD pathways and the PI3K/GSK3/SMAD1 pathway. In studies on the regeneration and degeneration of peripheral nerves, SMAD expression increased or decreased depending on the type of nerves used in experiments, research approach, method and degree of damage, and intervention strategy. Among SMADs, SMAD1 and SMAD4 were found to support nerve regeneration, whereas SMAD2 and SMAD6 were associated with neurodegeneration. Through further research, developing treatments that either promote the SMAD1/4 signaling pathway related to nerve regeneration or inhibit the SMAD2/6 signaling pathway related to nerve degeneration could greatly contribute to the treatment of nerve damage. Based on this study, in-depth research on the SMAD pathway related to nerve regeneration will be conducted to clearly understand the molecular mechanisms and establish effective treatments for nerve damage.

## Figures and Tables

**Table 1:** Review of the literature on SMAD expression after peripheral nerve injury.

Author/ Year/ Reference	Study Design	Species and/or Sample	Nerve/Injury Method	Detection Method	Target Substance(s) Associated with Autophagy	Results: Conclusions
Okuyama N et al., 2007 [12]	Animal study	Wistar rats	Hypoglossal nerve transection	RT-PCR, in situ hybridization, immunohistochemistry, Western blot analysis	*SMAD1*, -*2*, -*3*, -*4*, -*5*, -*6*, -*7*, -*8*	The expression of *Mad1*, -*2*, and -*4* mRNAs was significantly upregulated in injured motor neurons, whereas *SMAD8* mRNA was downregulated. *SMAD5–7* mRNA showed no significant alterations. : SMAD-mediated signaling might have an important role during nerve regeneration.
Fagoe ND et al., 2015 [13]	Animal study	Fisher 344 rats	Dorsal root transection injury	Immunohistochemistry, histological analysis, functional tests	ATF3, c-Jun, SMAD1, STAT3	Overexpression of ATF3, c-Jun, STAT3 and SMAD1 led to an increase in the rate of regeneration of injured dorsal root axons. : ATF3, c-Jun, STAT3 and SMAD1 individually contribute to regenerative axon growth of injured DRG neurons.
Finelli MJ et al., 2013 [14]	Animal study, in vivo	C57BL/6 mice, CD1 mice	Transection of sciatic nerve and ascending sensory fibers of spinal cord	DRG culture and neurite outgrowth assay, immunohistochemistry, chromatin immunoprecipitation, qRT-PCR, coimmunoprecipitation, Western blot, promoter sequence analysis	AcH4, SMAD1	AcH4 enrichment and pSMAD1 nuclear accumulation occurred predominantly in neurons and not glial cells in DRGs after a peripheral axotomy. : During the epigenetic reprogramming process, histone-modifying enzymes work together with SMAD1 to facilitate transcriptional regulation of RAGs.
Lee J et al., 2020 [15]	Animal study	*Prom1* KO mice	Sciatic nerve crush injury	Adult DRG cell culture, embryonic DRG cell culture, repleting assay	*Prom1*, SMAD2, cholesterol	PROM1 interacted with the TGF-βRI receptor, ALK4, to synergistically induce phosphorylation of SMAD2. : SMAD signaling is responsible for the enhanced axon regeneration induced by PROM1 overexpression.
Ying Si et al., 2013 [16]	Animal study, human ALS	G39A superoxide dismutase (SOD)1 mice, Human ALS muscle	Sciatic nerve transection	Behavioral assessment, Western blot, immunohistochemistry, statistical analysis, NGS, qRT-PCR	SMAD1/5/8	*SMAD8*, and to a lesser extent, *SMAD1* and -*5*, mRNAs were significantly elevated in human ALS muscle samples. *SMAD8* showed a substantially greater fold-change following sciatic injury compared with controls (up to 17-fold at end stage) relative to *SMAD1* and -*5* (up to 5-fold at end stage). : SMAD1, -5, -8 mRNA, and protein levels, as well as SMAD phosphorylation, are elevated in ALS muscle and could potentially serve as markers of disease progression.
Hata A et al., 1998 [17]	In vivo, in vitro	Xenopus embryos and in mammalian cells	Transfection with reporter plasmid and different amounts of SMAD6/CS2	In vivo phosphate labeling, yeast two-hybrid system, transcriptional assay, xenopus injections and animal cap assay, cloning of human and Xenopus SMAD6	SMAD6, BMP/SMAD1 pathway	BMP receptor signaling induced phosphorylation of SMAD1, which then associated with SMAD4. SMAD6 significantly blocked signaling by the BMP/SMAD1 pathway without interfering with receptor-mediated phosphorylation of SMAD1. : SMAD6 competes with SMAD4 for binding to receptor-activated SMAD1, yielding an inactive SMAD1–SMAD6 complex.
Parikh P et al., 2011 [18]	Animal study	C57BL/6 mice	Dorsal column transection, right sciatic nerve transection	AAV and intrathecal injection. Labeling of ascending sensory axons in the fasciculus gracilis	BMP/SMAD pathway	SMAD1 is developmentally regulated in DRG neurons and governs axon growth potential. BMP/SMAD1 signaling is essential for the conditioning effect in adult DRG neurons. Post-injury AAV-BMP4 injection promotes sensory axon regeneration. : Activation of SMAD1 promotes axonal regeneration following sciatic nerve injury.
Farrukh F et al., 2019 [19]	Animal study	Sprague Dawley rats	DC and SN crush injury	Microarray analysis, qRT-PCR, immunohistochemistry, BMP4 enzyme-linked immunosorbent assay, electrophysiology, functional testing	BMP4/SMAD1 pathway	The levels of *SMAD1*, *SMAD2*, *SMAD4*, *SMAD5*, *SMAD8* and *Bmp4 mRNA* were up-regulated 2.0- to 6.7-fold compared with sham controls, whereas Noggin was downregulated 2-fold. : Activation of the BMP4/SMAD1 pathway is a potential therapeutic strategy for promoting axon regenerative signaling in the CNS.
Kokubu N et al., 2018 [20]	Animal study, in vitro	Sprague-DawleySprague Dawley rats	Sciatic nerve crush injury	Functional assessment, immunohistochemical analysis, fluorescent double immunostaining, Western blot analysis, RT-PCR, SC culture, MTS cell proliferation assay	BMP-7, Noggin, SMAD, PTH (I-34)	BMP-7 and SMAD protein and mRNA were significantly upregulated in axon SCsSCs, and this increase was maintained for 4 weeks. Application of PTH(I-34) also upregulated BMP-7 on SCs. : Axonal regeneration can be induced by upregulating endogenous BMP-7 on SCs through PTH (I-34) administration.
Saijilafu et al., 2013 [21]	Animal study	CF-1 mice, bax−/− mice	Sciatic nerve crush and transection, cultured DRG neurons	qRT-PCR, in vivo electroporation, statistics, immunohistochemistry, fluorescence imaging	PI3K/GSK3-SMAD1	Acute depletion of the transcription factor SMAD1, which is induced by PI3K/GSK3 signaling, prevented axon regeneration in vivo in adult mice. : PI3K/GSK3/SMAD1 signaling is a central module for promoting sensory axon regeneration in the mammalian nervous system.
Thompson A et al., 2019 [22]	Animal study	Sprague Dawley rats, Fischer rats	Optic nerve crush injury, laser capture microdissection of retinal ganglion cell (RGC)	Immunocytochemistry, qRT-PCR, quantification of RGC survival and Muller cell activation	BMP4/SMAD1 pathway	*Bmp4*, *SMAD1*, *SMAD4*, *SMAD5*, *SMAD8*, *Smif*, and *Msg1* mRNAs were upregulated 3.2-, 4.5-, 5.5-, 2-, 3-, 3-, and 2-fold, respectively (*p* < 0.05), in RGCs with neurites compared with RGCs without neurites. : Activation of the BMP4/SMAD1 pathway promotes survival and axon regeneration independent of mTOR, and therefore may be of therapeutic interest.

Abbreviations: AAV, adeno-associated virus; AcH4, acetylated histone 4; ALS, amyotrophic lateral sclerosis; ATF3, activating transcription factor 3; BMP, bone morphogenic protein; DC, Dorsal column; DRG, dorsal root ganglion; DSC, dorsal spinal cord; GSK3, glycogen synthase kinase 3; NGS, next generation sequencer; NPC, neural precursor cell; ONC, Optic nerve crush; OPC, oligodendrocyte precursor cell; PI3K, phosphoinositide 3-kinase; PTH, parathyroid hormone; RAG, regeneration associated gene; RGC, retinal ganglion cell; SN, sciatic nerve; SC, Schwann cell; SCI, spinal cord injury; SOD, superoxide dismutase; STAT3, signal transducer and activator of transcription 3.

## Data Availability

The original contributions presented in the study are included in the article, further inquiries can be directed to the corresponding author/s.
